# Clinical Audits in a Postgraduate General Practice Training Program: An Evaluation of 8 Years' Experience

**DOI:** 10.1371/journal.pone.0043895

**Published:** 2012-09-10

**Authors:** Abeer Al-Baho, Maleka Serour, Adnan Al-Weqayyn, Mohammed AlHilali, Ali A. A. Sadek

**Affiliations:** 1 Department of Health Promotion, Ministry of Health, Kuwait City, Al-Kuwait, Kuwait; 2 Family Medicine Residency Program, Kuwait Institute for Medical Specialization, Kuwait City, Al-Kuwait, Kuwait; 3 Family Medicine Residency Program, Kuwait Institute for Medical Specialization, Kuwait City, Al-Kuwait, Kuwait; 4 Internal Medicine Department, Ain Shams University Hospitals, Cairo, Al-Qahirah, Egypt; 5 Department of Public Health, Faculty of Medicine Alexandria University, Alexandria, Al-Eskandariyya, Egypt; The University of Auckland, New Zealand

## Abstract

**Background:**

Clinical audit can be of valuable assistance to any program which aims to improve the quality of health care and its delivery. Yet without a coherent strategy aimed at evaluating audits' effectiveness, valuable opportunities will be overlooked. Clinical audit projects are required as a part of the formative assessment of trainees in the Family Medicine Residency Program (FMRP) in Kuwait. This study was undertaken to draw a picture of trainees' understanding of the audit project with attention to the knowledge of audit theory and its educational significance and scrutinize the difficulties confronted during the experience.

**Methodology/Principal Findings:**

The materials included the records of 133 audits carried out by trainees and 165 post course questionnaires carried out between 2004 and 2011. They were reviewed and analyzed. The majority of audit projects were performed on diabetic (44.4%) and hypertensive (38.3%) care. Regarding audits done on diabetic care, they were carried out to assess doctors' awareness about screening for smoking status (8.6%), microalbuminuria (19.3%), hemoglobin A1c (15.5%), retinopathy (10.3%), dyslipidemia (15.8%), peripheral neuropathy (8.8%), and other problems (21.7%). As for audits concerning hypertensive care, they were carried out to assess doctors' awareness about screening for smoking status (38.0%), obesity (26.0%), dyslipidemia (12.0%), microalbuminuria (10.0%) and other problems (14.0%). More than half the participants (68.48%) who attended the audit course stated that they ‘definitely agreed’ about understanding the meaning of clinical audit. Most of them (75.8%) ‘definitely agreed’ about realizing the importance of clinical audit in improving patients' care. About half (49.7%) of them ‘agreed’ that they can distinguish between ‘criteria’ and ‘standards’.

**Conclusion:**

The eight years of experience were beneficial. Trainees showed a good understanding of the idea behind auditing the services provided. They demonstrated their ability to improve the care given in health centers in which these projects were undertaken.

## Introduction

Clinical audit is recognized as one approach to improve the quality of patient care [1], [2], [3]. It has been increasing in importance for trainees in vocational training programs. Many departments of general practice in the UK currently incorporate audit as part of their course work. There is a clear existence of formal teaching of audits [4], [5]; however little is known about audit projects carried out by trainees in general practice. The FMRP in Kuwait has been aware of the importance of including the audit in the curriculum since 2000. The aim of the project is to introduce the trainees to their future responsibilities towards improving health services in the primary health care setting. A yearly course on “Clinical Audit Skills” was incorporated into the 2^nd^ year of the training program. Since then, an audit project is required for the purpose of formative assessment. A reassuring validity and reliability system and an eight criteria marking scale were used to assess trainees' projects [6]. These included reason for choice of audit, criterion/criteria chosen, standards set, preparation and planning, data collection “1”, changes to be evaluated, data collection “2” and conclusions. Each audit was marked independently by two examiners in the program before being successfully passed.

This study was undertaken to measure trainees' understanding of the audit project in terms of their grasped knowledge of audit theory, process, the audit's educational significance, as well as to examine difficulties that have been confronted during the whole experience.

The State of Kuwait is situated in the northeast of the Arabian Peninsula. The total area of Kuwait is 17,818 square Kilometers.

It is a high-income country and its economy ranks 10^th^ according to gross national income per capita published by the World Bank (2010).

Kuwait has a population of about 3,632,009 million of which about 1,164449 million are Kuwaiti citizens. Kuwait is divided into 5 health regions with 92 primary health care centers, representing the first level of service and these centers transfer patients to five general area hospitals. Thirty of these centers also function as training centers for the trainees under the supervision of 32 certified trainers in FMRP.

There are 789 general practitioners working in these centers, 301 of which are Board Certificated Family practitioners. However, the total number of Board Certificated family practitioners are 376; 60 have gone to work in the private sector and administrative departments, 10 have left to work abroad and 5 have resigned (data were obtained from the main statistical office of the FMRP).

Family Practice is a key element of all health care systems in Kuwait, and is recognized by health service providers as being of ever increasing importance.

Kuwait Institute for Medical specializations started the FMRP to train family physicians in 1983.

In 1987 the first batch of family physicians graduated from the program.

An examination and passing diploma certificate equivalent to that of the Membership of the Royal College of General Practitioners (MRCGP) examination was issued by the Royal College of General Practitioners In 1991.

An accreditation was awarded to the graduation certificate as MRCGP/International in 2005.

The FMRP consists of two phases. The first phase of the four year vocational training period starts with an introductory general practice period in January of each year, which lasts for seven months. This is followed by six attachments, alternating between general practice based training and hospital based training. Once this phase is completed, the candidate will be eligible to sit the first part of the MRCGP (International) exam, an applied knowledge test consisting of 200 single best answer questions.

The second phase is divided into six attachments, alternating between general practice based training and hospital based training, covering a period of 18 months. This is followed by six months of general practice based training, after which the candidate will be eligible to sit the final part of the MRCGP (INT) assessment, which consists of a Simulated Surgery (SS) exam, and a written modified essays questions paper.

Currently, there are 186 trainees from which 48 (28.6%) are male residents undergoing their training in the program.

## Materials and Methods

One hundred and thirty-three audit projects carried out by trainees in the FMRP were reviewed, in view of the diversity of health topics chosen, causes and rates of rejection of the submitted audits, and clinical improvement between the first and second data collections for each audit.

One hundred and sixty-five post course questionnaires, including six statements were revised to assess the effectiveness of the audit course during the previous 8 years. Questions on post course evaluation had the internal consistency reliability (Cronbach α) of 0.869 in the current study.

### Data Analysis

Each audit was examined with regard to the chosen topic, level of the performance at the time of the 1^st^ and 2^nd^ data collections, percentage achieved between the 1^s t^ and 2^nd^ data collections, expected standard for each audit, duration between the 1^st^ and 2^nd^ data collections, obstacles for each project and the frequency of rejection for each audit. Each of these were number coded. One hundred and sixty-five (collected from 8 batches who attended the course) were examined. The questionnaire consisted of 6 questions. All scale items were coded from a five-response Likert-type scale: 0 = Disagree strongly, 1 = Disagree, 2 = Unsure, 3 = Agree and 4 = Definitely Agree. The internal consistency of the questions was measured using Cronbach's α coefficient.

### Statistical methodology

Data were collected and coded then entered into an IBM compatible computer, using the SPSS version 20 for Windows. Entered data were checked for accuracy then for normality, using Kolmogorov-Smirnov & Shapiro-Wilk tests. Qualitative variables were expressed as number and percentage while quantitative variables were expressed as median and interquartile range (IQR).

The following statistical tests were used:

1. Independent samples Mann-Whitney's Z-test was used as a nonparametric test of significance for comparison between two sample medians.
2. The Kruskal-Wallis test (χ^2^-value) was used as a non-parametric test of significance for one-way comparison between more than two sample medians.
3. The Spearman's rank correlation coefficient (r) was used as a non-parametric measure of the mutual relationship between two not-normally distributed quantitative or ordinal variables.
4. Multivariate linear regression analysis for prediction of progress percentage.

A 5% level is chosen as a level of significance in all statistical significance tests used.

## Results

Table 1 shows the general characteristics of the audits done from 2004 to 2011.

**Table 1:** General characteristics of the audits done (2004–2011).

Variables		n	%
**Gender**	Female	90	67.7
	Male	43	32.3
**Topic of audit**	DM	59	44.4
	Hypertension	51	38.3
	Asthma	11	8.3
	Pediatrics	2	1.5
	Obesity	2	1.5
	Hyperthyroidism	7	5.3
	Dyslipidemia	1	0.8
**Progress**	3%–<10%	18	13.5
	10–<20%	20	15.0
	20–<30%	44	33.1
	≥30%	51	38.3
**Standard achieved**	No	125	94.0
	Yes	8	6.0
**Duration**	>3 months	1	0.8
	3–<6 months	58	43.6
	6–>12 months	55	41.4
	≥12 months	19	14.3

Most of the audit projects were performed on diabetic (44.4%) and hypertensive (38.3%) care.

All audit results showed improvement in the second data collection. The majority (71.4%) got an achievement above 20%. More than half of the audits were carried out over a duration longer than 6 months. Most (94.0%) didn't achieve the standard set for each audit.

Table 2 shows univariate analysis of the relationship of each of gender, topic and duration with the progress percentage. The achievement was significant (p value <0.001) for audits whose durations extended for longer than 6 months, while there was no significant association between resulting progress and either the trainees' gender or the chosen topic.

**Table 2:** Univariate analysis of the relationship between each of the trainee's gender, topic and project duration with the progress achieved as a percentage.

Variables		Progress							P-value
		3%–<10%	10%–<20%	20%–<30%	≥30%	Total	Median	IQR	
		(n = 18)	(n = 20)	(n = 44)	(n = 51)	(n = 133)	−27	−20	
**Gender**									0.358#
	Female	10	16	34	30	90	26.9	20.03	
	Male	8	4	10	21	43	28.7	21.8	
**Topic of audit**									0.833$
	DM	9	6	19	25	59	27.1	20.6	
	Hypertension	7	9	17	18	51	27	20.3	
	Asthma	1	3	4	3	11	24.3	16	
	Pediatrics	0	0	1	1	2	33.2	8.2	
	Obesity	0	1	0	1	2	29.8	25	
	Hypothyroidism	1	1	3	2	7	27.8	21.3	
	Dyslipidemia	0	0	0	1	1	38.8	0	
**Duration**									<0.001*
	>3 months	1	0	0	0	1	---	---	
	3–6 months	14	14	18	12	58	---	---	
	>6 months	3	5	21	26	55	---	---	
	one year	0	1	5	13	19	---	---	

# Mann-Whitney's test,

$ Kruskal Wallis Test and

* Spearman's rho Correlation.

Table 3 shows multivariate analysis of the relationship of each of gender, topic and duration with the progress percentage. There was neither significant association between progress and the topic chosen by the trainees nor their gender as shown earlier in Table 2. However, there was a significant increase in the progress of audits whose durations extended to more than 6 months (p = 0.024) and a highly significant association for those carried out for longer than one year (p = 0.008).

**Table 3:** Multivariate analysis of the relationship between each of the trainee's gender, topic and duration with the progress achieved as a percentage.

Progress = 4.349 (constant)	P-value
+2.11 (if the gender was male)	p = 0.345
−2.259 (if the topic was Hypertension)	p = 0.313
−4.696 (if the topic was Asthma)	p = 0.229
+2.962 (if the topic was Pediatrics)	p = 0.724
+6.908 (if the topic was Obesity)	p = 0.410
−5.857 (if the topic was Hypothyroidism)	p = 0.213
+5.394 (if the topic was Dyslipidemia)	p = 0.648
+17.488 (if the duration was 3–6 months)	p = 0.141
+26.947 (if the duration was 6–11 months)	p = 0.024
+32.179 (if the duration was one year)	p = 0.008

F_(10/122)_ = 3.73 P = 0.0002 R^2^ = 0.23.

Table 4 shows the result of 165 trainees' views on the course. Thirty-two trainees attended the course, and are currently performing the project but have not yet submitted them for assessment. More than half of participants (68.5%) stated that they ‘definitely agreed’ about understanding the meaning of clinical auditing. Most of them (75.8%) ‘definitely agreed’ about realizing the importance of clinical audit in improving patients' care. About half of them (49.7%) declared that they ‘definitely agreed’ that they can distinguish between ‘criteria’ and ‘standards’ and (40.6%) ‘agreed’ with the same distinction. By the end of the course, the majority ‘agreed’ that they can set realistic standards (70.3%) while 18.2% ‘definitely agreed’ about that. Most of them (60.6%) ‘agreed’ that they can choose suitable audit topics, while 26.7% ‘definitely agreed’ about that. More than half of them (55.8%) stated that they ‘definitely agreed’ that they understand the essential steps for conducting an audit cycle, while 40.6% ‘agreed’ with the same statement.

**Table 4:** Comparison between both groups (those who submitted and those who still haven't submitted their audits) according to their responses for the 6 post-course statements.

Statement	Groups	Definitely agree		Agree		Not sure		Disagree		P-value
		n	%	n	%	n	%	n	%	
**1: I can now understand the meaning of clinical audit**										
	1	87	65.4	45	33.8	1	0.8	0	0	0.105
	2	26	81.3	5	15.6	1	3.1	0	0	
	*Total*	113	68.5	50	30.3	2	1.2	0	0	
**2: I can realize the importance of clinical audit in improving patients' care**										
	1	97	72.9	33	24.8	3	2.3	0	0	0.099
	2	28	87.5	3	9.4	1	3.1	0	0	
	*Total*	125	75.8	36	21.8	4	2.4	0	0	
**3: I can distinguish between criteria and standards**										
	1	62	46.6	58	43.6	13	9.8	0	0	0.156
	2	20	62.5	9	28.1	3	9.4	0	0	
	*Total*	82	49.7	67	40.6	16	9.7	0	0	
**4: I can set realistic standards**										
	1	21	15.8	98	73.7	13	9.8	1	0.8	0.454
	2	9	28.1	18	56.3	5	15.6	0	0	
	*Total*	30	18.2	116	70.3	18	10.9	1	0.6	
**5: I can choose suitable audit topic**										
	1	28	21.1	87	65.4	15	11.3	3	2.3	0.004
	2	16	50	13	40.6	3	9.4	0	0	
	*Total*	44	26.7	100	60.6	18	10.9	3	1.8	
**6: I can now understand the essential steps for conducting an audit cycle**										
	1	68	51.1	60	45.1	5	3.8	0	0	0.019
	2	24	75	7	21.9	1	3.1	0	0	
	*Total*	92	55.8	67	40.6	6	3.6	0	0	

Mann-Whitney's Z test was used for comparing the two groups.

### Additional results

Eighty-seven audit project (65.4%) were successfully passed after the first submission, 34 (25.6%) after the second submission and 12 (9.0%) after the third submission.

Regarding audits done on diabetic care, they were carried out to assess doctors' awareness about screening for smoking status (8.6%), microalbuminuria (19.3%), hemoglobin A1c (15.5%), retinopathy (10.3%), dyslipidemia (15.8%), peripheral neuropathy (8.8%), and other problems (21.7%).

As for audits concerning hypertensive care, they were carried out to assess doctors' awareness about screening for smoking status (38.0%), obesity (26.0%), dyslipidemia (12.0%), microalbuminuria (10.0%) and other problems (14.0%).

Twenty-three candidates (24.1%) reported that they were faced by some technical barriers, such as difficult accessibility to laboratory facilities, frequent turnover of the practice team, negative attitudes of some clinicians, or lack of practical resources.

## Discussion

The main finding was that most of the audits were carried out on the care of patients with diabetes mellitus and hypertension. This may be explained by the high prevalence of these problems in Kuwait [7]–[10], as well as the availability of accurate disease registrations for these patients in family practice health centers, which facilitates the data collection process. This highlights the need for guidance in various aspects of patients' care which can be audited for both participants and leaders. This guidance would be useful for educators, professional associations, and medical certification bodies in planning, developing, implementing, evaluating, and supporting self-audit programs [11].

All audit projects were performed mainly on the area of processing the care. This can be explained by the fact that auditing aspects of the structure or the outcome of patients' care are far beyond the trainees' control. Lack of time is often cited as the main barrier of auditing the outcome, particularly within the time constraints of the training years.

One of the important findings of this study was that all audit projects showed some improvement in the care demonstrated at the time of the 2nd data collection, which added benefits to patient care and service delivery. This finding is consistent with other audit studies such as a national audit project on gynecological care done in Scotland [12] and one performed on general practice by medical students in Malaysia [13]. It emphasizes the value of including the audit project in the program.

More than half of the audits submitted were successfully passed after the first assessment. This reflects the good understanding of principles of auditing and in some way or another, also reflects the quality of training received throughout the training years.

The majority of audits didn't reach the agreed standards, in accordance with other studies [13]. Nevertheless, those with longer durations between the 1^st^ and 2^nd^ data collections showed better progress. This reinforces the suggestion to start the project as early as possible and have it initiated after completion of the clinical audit course. This will be enough time for the trainees to implement the required changes. In addition, soon after the course, trainees are enthusiastic about taking on the responsibility of the project.

Some barriers were mentioned by the trainees during the process. They pointed out that they implement the changes during summer periods, when most of the staff and patients were on annual leaves. Others stated that they had little time to make changes between the 1st and 2nd data collections, in accordance with a study [14] for trainees in a similar program in the West of Scotland region.

These re-enforce the recommendation of starting the project as early as possible to provide enough time to implement the required changes and to achieve better progress. Lack of practice resources was stated as another barrier, which is in accordance with other studies [15] done in the hospital setting. Negative attitudes of some clinicians towards auditing the provided care were cited by some trainees as one of the obstacles faced, which was consistent with other reports done in various countries by different medical disciplines [16]–[18]. This highlights the importance of ensuring all relevant staff is involved, improving multidisciplinary participation, and establishing the involvement of authorities and policy makers at crucial stages of the audit cycle. These all have the potential to improve the effectiveness of clinical audit programs as reported in previous studies [18], [19]. These studies reviewed the experiences of a wide assortment of clinicians, from medical consultants to professionals affiliated to medicine and healthcare.

Team training is also needed to deal with conflicts between individuals and those undertaking audits [18].

Analysis of the post course evaluation was positively encouraging. The majority stated that their understanding of the audit hypothesis and terminology had been enhanced, which was in accordance with other studies [14].

Most of the trainees realized at the end of the course the importance of clinical audit in improving patients' care in agreement with other reports [14]. They declared that they can set realistic standards as well as that they understood the audit steps.

A bigger portion of trainees who are currently performing their projects displayed more confidence in choosing a suitable audit topic and expressed a good understanding of the essential steps for conducting an audit cycle, in comparison with the trainees who have completed their projects. This was shown in the responses to the post course questionnaire. This may be explained by the program's increased experience with regard to audit as an educational tool, over the last 8 years.

Although most trainees stated that they can choose their audit topic, they still felt they needed support and guidance from their trainers, as they may face many barriers in collecting data and implementing the changes if the choice is inappropriate. Examples of these barriers are non-availability of accurate disease registration for the diverse health problems or the lack of evidence based criteria.

### Limitations of the study

More studies are needed to evaluate similar experiences done by the practice teams all over the area. This study only reflects trainees' performances and their views. Additional studies are needed to explore trainers' views of the experience as well as the views of the course tutors. This will refine and round off all aspects of the experience, and undoubtedly will help the implementation of the suggested recommendations.

## Conclusions

In conclusion, the eight years' experience showed many benefits, mainly improving the service provided to patients in the health centers in which these projects were carried out on. This was demonstrated by the progress achieved by all audits at the 2^nd^ data collection. The trainees demonstrated well their abilities to monitor and improve the quality of care by completing the audit cycle. According to our study, trainees in postgraduate programs who are working in the clinical setting can be of great support to the audit projects carried out in their workplaces.
